# Multiparametric Quantitative MRI in Neurological Diseases

**DOI:** 10.3389/fneur.2021.640239

**Published:** 2021-03-08

**Authors:** Alexander Seiler, Ulrike Nöth, Pavel Hok, Annemarie Reiländer, Michelle Maiworm, Simon Baudrexel, Sven Meuth, Felix Rosenow, Helmuth Steinmetz, Marlies Wagner, Elke Hattingen, Ralf Deichmann, René-Maxime Gracien

**Affiliations:** ^1^Department of Neurology, Goethe University, Frankfurt, Germany; ^2^Brain Imaging Center, Goethe University, Frankfurt, Germany; ^3^Center for Personalized Translational Epilepsy Research (CePTER) Consortium, Goethe University, Frankfurt, Germany; ^4^Department of Neurology, Palacký University Olomouc and University Hospital Olomouc, Olomouc, Czechia; ^5^Department of Neurology, Heinrich Heine University Düsseldorf, Düsseldorf, Germany; ^6^Epilepsy Center Frankfurt Rhine-Main, Center of Neurology and Neurosurgery, University Hospital, Frankfurt, Germany; ^7^Department of Neuroradiology, Goethe University, Frankfurt, Germany

**Keywords:** quantitative magnetic resonance imaging, neuroimaging, brain imaging, epilepsy, multiple sclerosis, neurodegeneration

## Abstract

Magnetic resonance imaging (MRI) is the gold standard imaging technique for diagnosis and monitoring of many neurological diseases. However, the application of conventional MRI in clinical routine is mainly limited to the visual detection of macroscopic tissue pathology since mixed tissue contrasts depending on hardware and protocol parameters hamper its application for the assessment of subtle or diffuse impairment of the structural tissue integrity. Multiparametric quantitative (q)MRI determines tissue parameters quantitatively, enabling the detection of microstructural processes related to tissue remodeling in aging and neurological diseases. In contrast to measuring tissue atrophy via structural imaging, multiparametric qMRI allows for investigating biologically distinct microstructural processes, which precede changes of the tissue volume. This facilitates a more comprehensive characterization of tissue alterations by revealing early impairment of the microstructural integrity and specific disease-related patterns. So far, qMRI techniques have been employed in a wide range of neurological diseases, including in particular conditions with inflammatory, cerebrovascular and neurodegenerative pathology. Numerous studies suggest that qMRI might add valuable information, including the detection of microstructural tissue damage in areas appearing normal on conventional MRI and unveiling the microstructural correlates of clinical manifestations. This review will give an overview of current qMRI techniques, the most relevant tissue parameters and potential applications in neurological diseases, such as early (differential) diagnosis, monitoring of disease progression, and evaluating effects of therapeutic interventions.

## Introduction

Conventional magnetic resonance imaging (MRI) techniques are used in the clinical routine to diagnose and monitor neurological diseases (1). However, clinical routine MRI of the brain primarily visualizes macroscopic lesions. In contrast, it is difficult to assess diffuse or subtle changes with conventional MRI or underlying pathological principles because it shows mixed contrasts which depend on protocol settings and hardware parameters such as the magnetic field strength and inhomogeneities of the static magnetic field and radiofrequency coil sensitivities (2).

**Figure 1:**
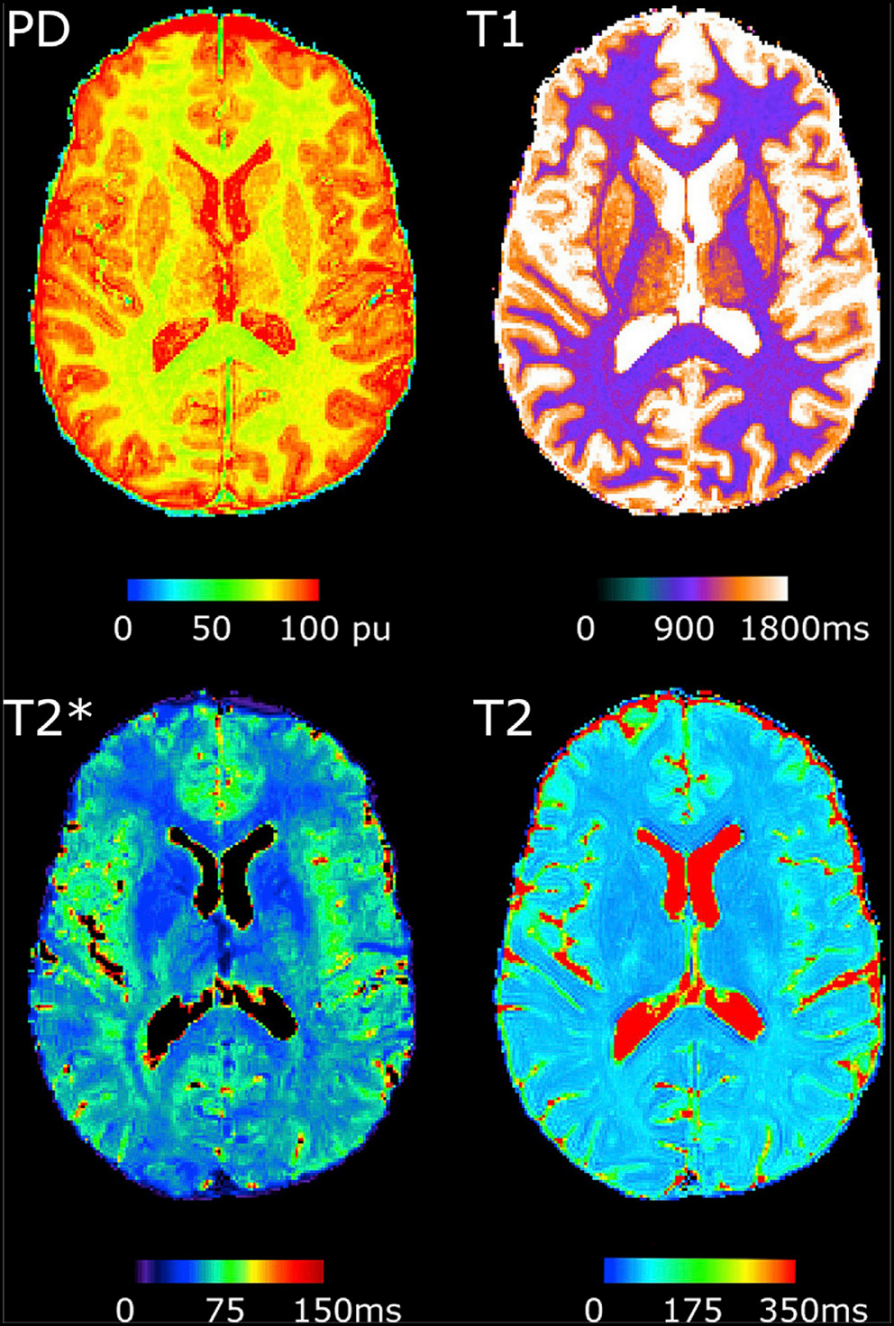
Examples of quantitative MRI maps of a single subject. PD, Proton density. The figure has been published under a Creative Commons license (https://creativecommons.org/licenses/by-nc-nd/4.0/) by Gracien et al. (3).

In contrast, quantitative MRI (qMRI) techniques measure actual tissue parameters such as the T1-, T2-, T2*-relaxation times or the proton density (PD), largely eliminating hardware effects (2). Figure 1 demonstrates examples of qMRI maps. The respective parameter maps provide quantitative parameter values for each single voxel, which can be compared between groups, study centers and in longitudinal investigations. Accordingly, the values can be used to assess diffuse and subtle changes in tissue composition in neurological diseases. QMRI measurements are reproducible with only a small scan-rescan deviation of around 1% for state–of-the-art MR scanners, whereas deviations between different scanner models are around 3.5% (3). However, systematic differences between scanners can be quantified with phantom measurements, allowing for *post-hoc* corrections. Furthermore, a small inter-site bias as well as small inter-site and intra-site coefficient of variation was observed for multiparametric mapping approaches with relatively short acquisition times, demonstrating the potential of these methods for multi-center investigations (4, 5). While other somewhat more specific MRI techniques such as perfusion mapping using arterial spin-labeling (ASL) also allow for the quantitative characterization of certain perfusion properties (6–8), this review mainly focuses on quantitative relaxometry and PD mapping. These increasingly applied imaging techniques allow for the multiparametric assessment and characterization of microstructural tissue alterations and biochemical processes related to neurological diseases of different etiologies. The main objective of this review is to give a compact but comprehensive overview of current and future potential applications for multiparametric qMRI in neurological diseases, including the tissue parameters, which are most relevant for investigating the respective diseases, given the underlying pathophysiology and pathological changes of tissue microstructure, and potential applications in research and clinical practice. Furthermore, we summarize the major current limitations of qMRI techniques, the main technical issues to be solved for further improvement and applications of qMRI techniques for research and clinical purposes.

## What Can Be Measured With qMRI?

qMRI techniques measure physical tissue parameter values. These values depend on the microstructural tissue composition. However, most qMRI parameters depend on various microstructural processes and the relative proportions of different tissue compartments. In contrast to conventional MR imaging used in clinical routine, most qMRI techniques require the acquisition of a series of weighted raw images with different timing parameters such as different echo or inversion times, allowing for exponential fitting of the signal time course and yielding maps of the respective parameter. The resulting acquisition times can be relatively long, depending on the number of images in the series. As an example, T2 mapping with a high number of different echo times allows for the distinction between different tissue fractions, e.g., the quantification of the myelin water content, but requires long experiment durations. Still, the reliable detection of significant differences in qMRI parameters between a group of patients and a healthy population may already be feasible with a low number of echo times and thus a shorter acquisition time. While the parameter PD measures free tissue water (9, 10), T1 values are positively correlated with water content and gliosis and negatively correlated with iron and myelin content (10, 11). The Magnetization Transfer Ratio (MTR) is sensitive to the macromolecular content and the fraction of bound water and commonly used as surrogate marker of myelin (9). T2 is increased by the free water fraction but shortened by myelin-bound water (12) and iron deposition (13). T2* is a marker for the iron content (10, 14). Furthermore, demyelination is known to prolong the T2* relaxation time (15). Multiparametric qMRI approaches can help to better understand and characterize disease related tissue remodeling and damage in neurological diseases. Therefore, multiparametric mapping protocols have been proposed for the simultaneous deduction of several parameter maps reflecting multiple tissue properties and microstructural attributes (4, 10).

## Multiparametric qMRI in Multiple Sclerosis (MS)

Autoimmune inflammation in MS causes cerebral MS lesions, resulting in clinical symptoms such as the relapses in relapsing-remitting MS (RRMS). However, macroscopic lesions are known to be only the “tip of the iceberg” of tissue damage in MS (16). Histopathological studies revealed that gliosis, demyelination, axonal loss, and infiltration of cells of the immune system also occur in tissue which appears normal on conventional MRI in MS (17, 18). QMRI allows for the quantification of these microstructural changes in tissue composition induced by MS. Multiparametric qMRI analyses of MS patients revealed increased T1, T2, T2*, PD, and decreased magnetization transfer ratio (MTR) values in normal-appearing white matter (WM) and cortical gray matter (GM) (19–22). A previous multiparametric qMRI study utilizing surface-based analysis techniques observed spatially inhomogeneous cortical T2-, T2*-, and PD-increases with parameter-specific spatial distribution patterns indicating that in some cortical regions certain microstructural changes (such as an increased water content) might dominate, while in other cortical regions other processes might be more prominent in MS patients (22). Importantly, qMRI parameter changes in normal-appearing tissue were already observed in early stages of the disease (23–25). Furthermore, several studies reported a correlation between qMRI parameter values, indicating diffuse changes in tissue composition in MS, and the clinical/cognitive status, thus demonstrating the relevance of diffuse cerebral processes for patients (22, 26).

Longitudinal qMRI studies in MS have the potential to demonstrate the ability of qMRI techniques to assess tissue remodeling over time. However, two longitudinal investigations reported no longitudinal changes of mean T1 values in normal-appearing WM or GM in MS (23, 27). The longitudinal study by Parry et al. (28) observed decreasing GM T1 values, while a different investigation reported increasing cortical PD- and T1-values (29). Future longitudinal MS studies are required which utilize advanced data analysis methods minimizing potential sources of bias such as partial volume effects to probe the potential of multiparametric qMRI techniques for longitudinal study designs in MS.

## Multiparametric qMRI Applications in Epilepsy

Epilepsy is a disease of the brain characterized by an enduring predisposition to generate epileptic seizures (30). The identification of underlying epileptogenic lesions in patients diagnosed with structural epilepsy can be challenging. MRI techniques can help to detect focal cerebral lesions in epilepsy patients and to quantify diffuse disease-related changes in brain tissue. Bernasconi reported that T2 relaxometry can quantify hippocampal damage in temporal lobe epilepsy (TLE) even in patients with normal conventional MRI and correctly identify the side of the hippocampal changes in most of the patients (31). A more recent study employed a multiparametric qMRI approach including T1/T2 mapping and diffusion tensor imaging (DTI) techniques combined with support vector machines to distinguish TLE patients from healthy control subjects (32). The accuracy amounted to 88.9%. Furthermore, a prolonged T2 relaxation time was observed in the amygdala and hippocampus for epilepsy patients with suspected limbic encephalitis, whereas the expected increase of the signal intensity in T2-weighted datasets was not found (33).

Another potential application of qMRI is the detection of focal cortical dysplasia (FCD), a highly epileptogenic structural cerebral lesion often causing therapy-refractory epilepsy. The standard methods for improved visualization of FCD described by Kassubek et al. (34) and Huppertz et al. (35) are based on conventional MRI datasets. However, these methods require mechanisms for intensity normalization/bias-field correction. QMRI techniques might be advantageous for FCD detection since the parameter maps are (intrinsically) corrected for any hardware bias (2). Nöth et al. (36) developed a method for improved FCD visualization, utilizing quantitative T1 maps, and assessing the cortical extent and the smoothness of the WM-GM-junction. Furthermore, a different method for improved FCD visualization based on multiparametric qMRI data (T1, T2, and PD maps) and surface-based analysis techniques has been proposed, measuring the standard deviations of the respective parameters at the WM-cortex junction (37). The resulting information on junction smoothness and additional measures of the local cortical thickness are then used in combination to artificially enhance the signal in conventional fluid attenuated inversion recovery (FLAIR) datasets, thus highlighting regions suspicious of FCD. Future studies are required to evaluate the clinical relevance of qMRI in epilepsy more closely. Consequently, as demonstrated by the methods applied for the detection of FCD in epilepsy, complementing conventional MRI by qMRI allows for an improved visualization of FCD, thus facilitating a task which is one of the major challenges in the diagnosis of epilepsies of unknown origin with MRI. Important challenges are the detection of previously undetected epileptogenic lesions and the discovery of qMRI biomarkers of epileptogenicity and the seizure onset zone. QMRI may also allow to investigate the contributions of blood-brain barrier dysfunction (38) and neuroinflammation to initiation and maintenance of epileptogenesis and related cognitive dysfunction (39).

## qMRI in Cerebrovascular Disease

Chronic hemodynamic compromise due to steno-occlusive disease of the brain-supplying vasculature increases the risk for ischemic stroke (40, 41). Furthermore, cerebral hypoperfusion is associated with cognitive impairment, even in subjects for whom conventional MRI provides no evidence of acute or chronic ischemic lesions (42). Cerebral changes in tissue composition such as microglial activation and demyelination were reported in experimental studies on cerebral hypoperfusion (43). Since these pathological tissue alterations are detectable with qMRI, an application of these techniques in patients with cerebrovascular disease can be expected to be a promising approach. In fact, significant increases of T2 values were observed in normal-appearing WM and normal-appearing cortical GM affected by chronic hypoperfusion in patients with unilateral high-grade carotid-occlusive disease (44, 45). The relative prolongation of the T2 relaxation time correlated with the degree of hemodynamic impairment, indicating that T2 mapping detects gliotic tissue conversion, demyelination and enlargement of the extracellular compartment related to local hypoperfusion (44, 45). In addition, changes of T2 values seem to precede macroscopic tissue atrophy (44). Therefore, the T2 relaxation time might be a useful imaging biomarker of early and subtle hypoperfusion-related microstructural tissue damage. A further important application of qMRI in chronic cerebrovascular diseases concerns the characterization of microstructural damage in normal-appearing WM in cerebral small vessel disease (SVD). Previous investigations found significant increases of T2 values within the cerebral WM surrounding WM hyperintensities in SVD patients, potentially reflecting microstructural gliosis, demyelination, axonal damage and increased tissue water content in these areas (46). Furthermore, due to its sensitivity to the tissue fluid content, the T1 relaxation time seems to be a promising imaging biomarker with regard to the detection of blood-brain barrier disruption as a key mechanism of microstructural WM damage in SVD (47, 48). It remains to be clarified whether these parameters may be suitable imaging biomarkers of pathological tissue remodeling underlying common clinical manifestations of SVD such as cognitive decline or gait disturbances. In acute ischemic stroke, an important possible domain of qMRI is the determination of the time of stroke occurrence in patients with wake-up stroke or in patients with severe aphasia and unwitnessed symptom onset. Because of the immediate implications for the therapeutical management, this is highly relevant. So far, T2 mapping has been employed in clinical pilot studies, revealing promising significant associations between T2 values in the ischemic core and time from symptom onset (49–51). Future studies are required to confirm the validity of qMRI parameters as indicators of the time since symptom onset and to assess their reliability across different scanners and imaging protocols. In summary, qMRI covers numerous potential applications in cerebrovascular diseases with a realistic translation into standard patient care, resulting in possible consequences with regard to acute treatment and clinical management of neurological patients.

## Multiparametric qMRI in Aging and Neurodegeneration

Given the steadily increasing life expectancy and the increasing prevalence of neurodegenerative diseases manifesting at higher age in the Western societies, the differentiation between normal aging and early stages of neurodegeneration is crucial to identify individuals at risk of cognitive decline. In order to capture cases in which the process of aging is to a certain degree accelerated or altered by the detrimental impact of various factors on the microstructural integrity of the brain tissue, it is important to define and characterize microstructural tissue alterations associated with normal aging. Numerous distinct tissue-remodeling processes on the microstructural level are associated with physiological aging. Previous studies employing multiparametric qMRI consistently demonstrated decreasing T1 values and increasing T2 values with increasing age in the adult lifespan for both GM and WM (10, 52–57). Besides a decreasing overall tissue water fraction and a regression of dendrites, age-related shortening of T1 has been attributed to an increasing tissue iron deposition, which seems to be a prominent process in brain aging (58). Increasing T2 values have been mainly interpreted as the result of demyelination taking place with increasing age (49). Concerning quantitative T2*, the directionality of age-related changes seems to depend on the tissue type and local microstructural properties (59, 60). In more detail, while in deep GM structures a negative association between T2* values and age was observed, T2* values and age were positively correlated in WM (60). This discrepancy may be explained by an age-related iron accumulation in deep GM nuclei leading to a decrease of T2*, while prominent demyelination in WM may lead to a prolongation of T2* due to inherent T2 (spin-spin) effects (59, 60). The bidirectional influences of distinct age-related processes on T2* may be the reason for the lack of a correlation between T2* values and age in a recent study investigating age-related changes of the cortical GM microstructure with multiparametric qMRI (59). In contrast, the reversible transverse relaxation time T2′ (calculated as 1/T2′ = 1/T2*-1/T2), which is corrected for spin-spin effects, showed widespread negative associations with age across the cerebral cortex, most likely due to an age-related cortical iron deposition occurring during physiological aging (59). Concerning possible applications of qMRI in pathological neurodegeneration, several studies suggested that the T2 relaxation time might be a promising imaging biomarker for differentiating between cognitively normal elderly subjects and individuals with mild cognitive impairment (MCI) or Alzheimer's disease (AD) (61–64). Furthermore, the T2 relaxation time seems to be able to discriminate between cases of MCI and AD within a population of cognitively impaired elderly individuals, which is an important finding from a clinical point of view (61–63). Despite revealing a regionally heterogeneous pattern of qMRI parameter changes in subjects with cognitive complaints, a recent multiparametric qMRI study suggested that also T1 and T2* mapping may be useful to detect microstructural tissue alterations in MCI patients compared to healthy controls (65). In Parkinson's disease, a widely disseminated application of qMRI techniques is the evaluation of brain iron accumulation and neuronal loss in the dopaminergic areas of the brainstem as well as in other basal ganglia structures (66–68). For this purpose, especially T2, T2*, and T1 values were determined in previous studies (69, 70). Furthermore, qMRI may provide insights into cortical tissue pathology in patients with Parkinson's disease. So far, especially T1 relaxometry was applied for the investigation of cortical involvement (71, 72), in particular for studying the cortical spread-out of the neurodegenerative process and its relation to cognitive decline (72). Furthermore, some studies have suggested the suitability of T2 and T2′ mapping for differentiating between different causes of Parkinsonian syndromes (73, 74), thus potentially adding highly relevant information from a clinical point of view.

## Discussion

Multiparametric qMRI provides a set of parameters, which allow for gaining deeper insights into microstructural tissue alterations and impairment of microstructural integrity in a range of neurological diseases and in physiological aging. Although most of these parameters are not specific for a single distinct microstructural process, predominant sensitivities to certain aspects of microstructural tissue pathology enable the comprehensive characterization of disease- or age-related tissue changes. The utility of qMRI in this regard can be further increased if results from preclinical, experimental and histological studies are taken into account and are correlated with findings from qMRI investigations. One of the most important advantages of multiparametric qMRI is the detection of subtle impairment of the microstructural tissue integrity, which can accrue before measurable tissue volume reduction occurs (44, 59). Therefore, apart from depicting distinct biologically relevant mechanisms of tissue remodeling, qMRI techniques allow for investigating microstructural processes, which both potentially underlie and precede measurable tissue atrophy (44). A recent study demonstrated the stability of qMRI results, showing low intra-scanner deviations in repeated measurements and a high reproducibility of qMRI findings across different scanner models (3). Thus, besides the high utility of qMRI for detecting disease-related tissue changes in patients with neurological diseases compared to healthy control subjects in single center studies, these findings also suggest a high reliability of qMRI techniques for longitudinal and multicenter studies (3). The combination with recent advances in hardware development will help to further improve the precision of multiparametric qMRI for the characterization of pathological changes in tissue microstructure in neurological diseases. For instance, the implementation of qMRI sequences on 7T MRI scanners, which are increasingly used in clinical research, will result in qMRI parameter maps with a higher spatial resolution. It has to be taken into account that the relaxation times (T1, T2, T2*) depend on the field strength. However, this does not affect the comparability of parameter maps acquired on different scanners, provided the field strength is identical. Increasing the spatial resolution for T2-, T2*-, and T2′-mapping improves the spatial alignment with other structural datasets during coregistration procedures and reduces partial volume effects. However, increased spatial resolution and a high number of echoes make the qMRI maps more prone to artifacts resulting from intra-scan subject motion, because of the longer acquisition durations, which may be disadvantageous in patients with (acute) neurological diseases. It should be noted that qMRI techniques can only assess biological and biochemical processes which have an impact on the tissue microstructure. This limits their usefulness in neurological diseases which are merely characterized by a pathological alteration of the functional connectivity without any consequences on the brain microstructure, as may be the case for some movement disorders such as dystonia (75). Future studies on neurological diseases might benefit from investigating the interaction between functional properties (such as functional connectivity or brain perfusion) and microstructural tissue changes that can be assessed by multiparametric qMRI. Furthermore, while many of the multiparametric qMRI findings in the different neurological diseases described above are plausible and can be interpreted from a pathophysiological point of view, a histological validation—e.g. obtained from appropriate and disease-specific animal models—is still lacking in many cases. Therefore, researchers in the field of multiparametric qMRI should aim at correlating qMRI and histological findings in neurological diseases whenever possible. Future research on qMRI methods should focus in particular on reducing acquisition times and improving motion correction algorithms. This will further enhance the utility of multiparametric qMRI for the investigation of neurological diseases, also with regard to supporting diagnoses and important clinical tasks such as monitoring disease progression and effects of therapeutical interventions. Given the relevance of these tasks, more longitudinal studies using multiparametric qMRI in neurological diseases are warranted and needed. Furthermore, it would be a great step forward if more scanner vendors included qMRI techniques in the set of release sequences. Recently developed open-source software packages for qMRI data processing and quantitative parameter mapping, including tools for image coregistration and tissue segmentation, will help to further facilitate the use of qMRI methods, both in research and clinical applications (4, 76, 77).

## Author Contributions

AS and R-MG wrote the first draft of the manuscript. All authors reviewed the manuscript, contributed to the manuscript revision and approved the submitted version.

## Conflict of Interest

EH has received speaker's honoraria from BRACCO. FR has received honoraria for presentations and consultations from Arvelle Therapeutics, EISAI, UCB Pharma, Novartis Oncology, Medtronic, GW-Pharmaceuticals, as well as research grants from UCB, European Union, Deutsche Forschungsgemeinschaft, European Science Foundation and the Hessonian Ministries of Science and Arts and of Social Affairs and Integration. HS has received speaker's honoraria from Bayer, Sanofi and Boehringer Ingelheim. The remaining authors declare that the research was conducted in the absence of any commercial or financial relationships that could be construed as a potential conflict of interest.

## Abbreviations

DTI: diffusion tensor imaging
FCD: focal cortical dysplasia
GM: gray matter
MRI: magnetic resonance imaging
MS: multiple sclerosis
MTR: magnetization transfer ratio
NA: normal-appearing
PD: proton density
qMRI: quantitative MRI
RRMS: relapsing-remitting MS
SVD: small vessel disease
TLE: temporal lobe epilepsy
WM: white matter.
